# Severe vaso-occlusive lupus retinopathy: Purtscher-like retinopathy: a case report

**DOI:** 10.1097/MD.0000000000050535

**Published:** 2026-09-04

**Authors:** Kaiyao Chi, Xiaoyang Xie, Tong Guo, Daoxi Lei, Huili Li

**Affiliations:** a Department of Ophthalmology, The First Affiliated Hospital of Chongqing University of Chinese Medicine, Chongqing, China; b School of Ophthalmology, Chengdu University of Traditional Chinese Medicine, Chengdu, China; c College of Traditional Chinese Medicine, Chongqing University of Chinese Medicine, Chongqing, China.

**Keywords:** lupus retinopathy, Purtscher-like retinopathy, systemic lupus erythematosus, traditional Chinese medicine

## Abstract

**Rationale:**

Purtscher-like retinopathy is an extremely rare, vision-threatening microvascular complication of systemic lupus erythematosus (SLE). To date, there is no standardized, unified treatment guideline for this condition worldwide, and conventional interventions including systemic glucocorticoids, immunosuppressants, and anti- vascular endothelial growth factor therapy often yield unsatisfactory visual outcomes. Additionally, the early ocular manifestations of SLE are easily overlooked due to atypical systemic symptoms, leading to delayed diagnosis and irreversible visual impairment.

**Patient concerns:**

A 24-year-old female developed blurred vision after coronavirus disease 2019 infection, which was initially neglected. She subsequently presented with persistent fever, lymphadenopathy, recurrent seizures and altered consciousness, and was diagnosed with SLE complicated by neuropsychiatric lupus. Her visual loss progressively aggravated during systemic treatment.

**Diagnoses:**

Systemic workup confirmed active SLE with neuropsychiatric involvement. Serial ophthalmic examinations revealed bilateral Purtscher-like retinopathy characterized by venous tortuosity, extensive cotton-wool spots, retinal hemorrhages, macular edema, diffuse capillary non-perfusion, and foveal arteriovenous anastomosis, with secondary right eye vitreous hemorrhage.

**Interventions:**

On the basis of standard SLE treatment with glucocorticoids and immunosuppressants, comprehensive interventions were implemented, including blood pressure optimization, anticoagulant discontinuation to mitigate bleeding risk, fractional panretinal photocoagulation for retinal ischemia, and adjunctive modified Ditan Decoction based on Traditional Chinese Medicine syndrome differentiation.

**Outcomes:**

During follow-up, blood pressure was gradually stabilized. Right eye vitreous hemorrhage resolved completely without recurrent bleeding. Retinal ischemia improved after photocoagulation, with partial regression of foveal arteriovenous anastomosis. Visual acuity improved modestly and remained stable at the final assessment.

**Lessons:**

Ocular microvascular lesions may precede systemic disease flares in SLE, underscoring the necessity of routine ophthalmologic screening for newly diagnosed patients, particularly those with neuropsychiatric involvement. Adjunctive Traditional Chinese Medicine therapy may offer incremental benefit for refractory Purtscher-like retinopathy, representing a complementary strategy that warrants further investigation.

## 1. Introduction

Systemic lupus erythematosus (SLE) is a chronic, systemic autoimmune disease driven by pathogenic immune complex deposition and autoantibody-mediated vasculitis.^[1]^ It exhibits a striking female predominance, predominantly affecting women of reproductive age, with a reported female-to-male incidence ratio of approximately 9:1.^[2]^ The clinical spectrum of SLE spans multiple organ systems, most notably the skin, kidneys, and central nervous system (CNS), and its severity characteristically fluctuates with alternating phases of flare and remission.^[3]^ Although a relatively comprehensive appreciation of the systemic manifestations of SLE had emerged by the mid-to-late 19th century, ocular complications were not recognized as a clinically significant dimension of the disease until the 1930s.^[4]^

Ocular manifestations of SLE, including dry keratoconjunctivitis, anterior uveitis, and lupus retinopathy, are now identified in more than one-third of patients.^[5,6]^ Among these, lupus retinopathy, though rare, poses a substantial threat to vision and frequently accompanies flares of lupus nephritis or CNS involvement.^[7,8]^ The pathogenesis centers on the deposition of immune complexes within the retinal microvasculature that supplies the nerve fiber layer.^[9]^ This deposition initiates an immune cascade, triggering the release of neutrophil lysosomes and reactive oxygen species, which in turn cause endothelial injury, increased vascular permeability, and extravasation of plasma proteins and cellular elements. These events culminate in widespread inflammation and secondary vascular occlusion.^[9]^ On color fundus photography, the hallmark finding is the presence of cotton-wool spots, arising from precapillary arteriolar obstruction in the retina. These lesions may appear singly or as scattered multiples, and they often coexist with retinal hemorrhages and vascular tortuosity, further highlighting the complexity of this condition.^[10,11]^

Purtscher-like retinopathy is a particularly rare retinal manifestation in the spectrum of SLE. First described by Otmar Purtscher in 1910, Purtscher retinopathy classically presents with sudden vision loss following severe head trauma.^[12]^ The characteristic funduscopic findings include Purtscher flecks and cotton-wool spots confined to the posterior pole, often accompanied by mild to moderate retinal hemorrhages.^[13]^ Although the precise pathogenesis remains incompletely understood, proposed mechanisms include immune complex microembolism, leukocyte embolism, capillary endothelial injury, altered intracranial pressure, and hyperviscosity, which collectively precipitate precapillary arteriolar occlusion and microvascular infarction within the retinal nerve fiber layer.^[14]^ Beyond head trauma, Purtscher retinopathy has been associated with other traumatic events such as long bone fractures, head or chest compression, and crush injuries. Notably, identical funduscopic changes have been documented in various nontraumatic conditions, including acute pancreatitis,^[15]^ hemolysis, elevated liver enzymes, low platelet count syndrome,^[16]^ and multiple myeloma,^[17]^ giving rise to the term “Purtscher-like retinopathy.” A shared funduscopic hallmark of both Purtscher retinopathy and Purtscher-like retinopathy is the presence of Purtscher flecks: multiple polygonal zones of retinal whitening situated between arterioles and venules, frequently accompanied by superficial cotton-wool spots at the posterior pole. These flecks are distinct from the confluent retinal whitening seen in proximal retinal artery occlusion and from the superficial, feathery, irregularly bordered cotton-wool spots that arise from distal capillary occlusion.

## 2. Case description

This article presents a unique case of a patient with SLE exhibiting retinal manifestations, with blurred vision serving as an initial, albeit overlooked, symptom by the patient.

A 24-year-old female patient sought admission to the rheumatology and immunology department of an external institution due to persistent fever lasting one month. She denied any prior history of refractive errors, hypertension, or renal pathology. Examination revealed bilateral cervical lymphadenopathy, with puncture biopsy showing lymphoid tissue exhibiting extensive necrosis and inflammatory changes. Laboratory tests indicated abnormal antinuclear antibody levels, a positive anti-double-stranded DNA antibody test, a negative antiphospholipid IgG test, slightly elevated urine protein levels, leukopenia, lymphocytopenia, and thrombocytopenia. Despite the administration of methylprednisolone at doses of 40 mg/day and subsequently 1 mg/kg, the patient’s fever persisted.

Two weeks into her hospital stay, she experienced frequent seizures and disturbances of consciousness. Cerebrospinal fluid examination yielded negative results, while an enhanced cranial magnetic resonance imaging scan revealed slight thickening and enhancement of the tentorium cerebelli. She was rescued with an intensive multi-modal regimen comprising tracheal intubation and mechanical ventilation, intravenous immunoglobulin combined with glucocorticoids, antiepileptic therapy (valproic acid, midazolam, and propofol), glycerol fructose for intracranial pressure reduction, vasopressor support with epinephrine and metaraminol to maintain organ perfusion, piperacillin-tazobactam for infection management, low-molecular-weight heparin for anticoagulation, blood transfusion to correct anemia, and enteral nutrition. Following these interventions, the seizures and impaired consciousness resolved.

However, the patient reported severe vision loss. In retrospect, blurred vision had actually preceded the onset of fever, having first appeared after a coronavirus disease 2019 infection in late 2022, which led the patient to disregard these symptoms at presentation. Prior to her discharge, a comprehensive fundus examination was conducted, revealing the presence of macular edema. Consequently, a treatment regimen consisting of methylprednisolone, mycophenolate mofetil, and rivaroxaban was initiated (baseline).

Upon discharge from a prior hospitalization, the patient sought medical attention at our institution due to persistent visual blurring (first visit). Initial anthropometric and physiological assessments revealed a height of 157 cm, a weight of 71 kg, and a markedly elevated blood pressure (BP) of 182/115 mm Hg. Visual acuity was 0.04 in the right eye and 0.02 in the left eye, with no improvement following refractive correction, indicating severe visual impairment. Bilateral ocular examination further identified minimal hemorrhagic flocculent vitreous opacity. Funduscopic evaluation unveiled features consistent with Purtscher-like retinopathy, including tortuous and dilated retinal veins, white sheathing of blood vessels, multiple patchy hemorrhages, cotton-wool spots, and venous beading alterations.

**Figure 1.**
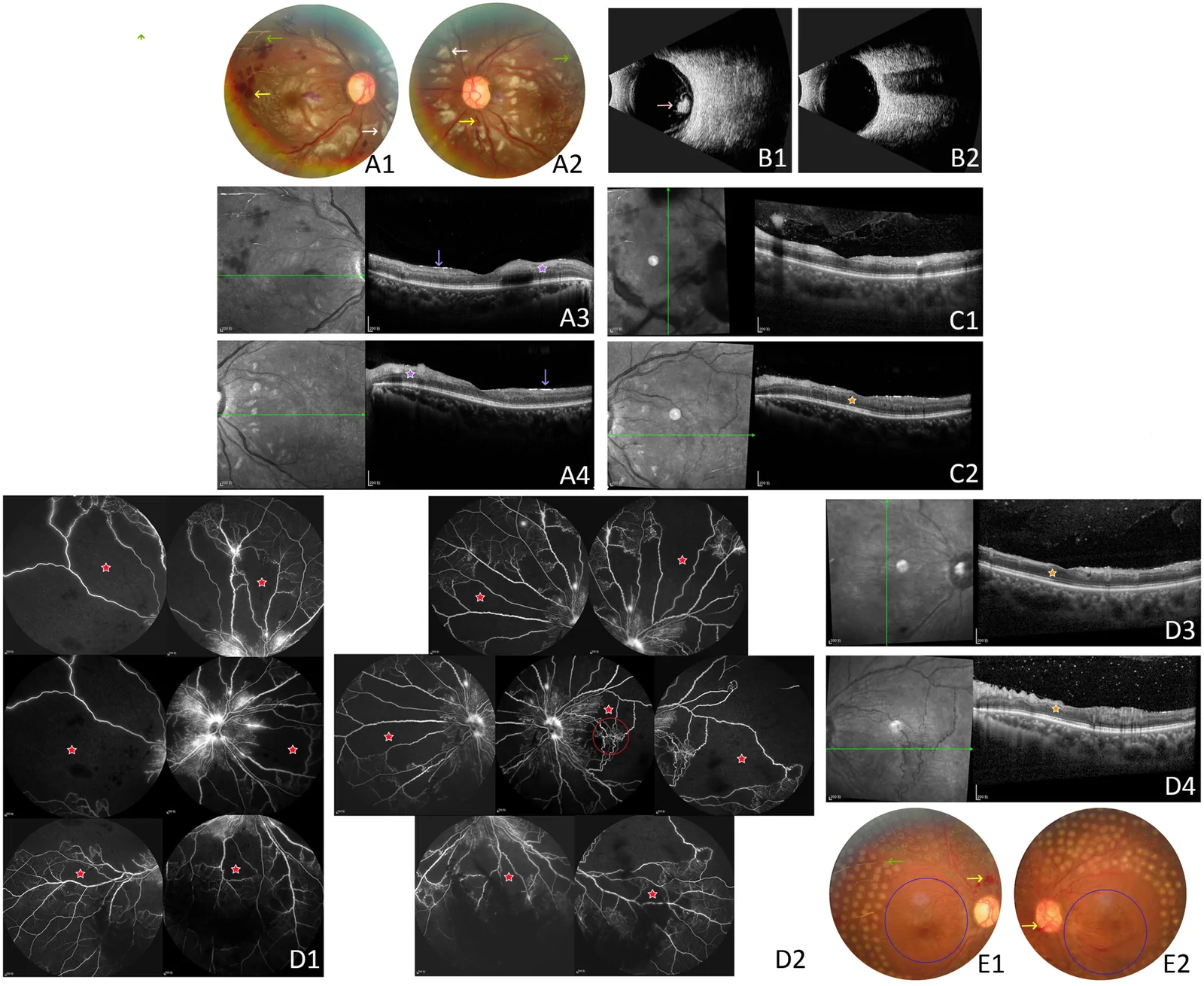
Sequential ocular manifestations in the patient. (A) (Initial diagnosis): Fundus photograph showing Purtscher flecken (white arrow), intraretinal hemorrhages (yellow arrow), and vascular sheathing (green arrow). Macular OCT reveals epiretinal hyperreflective membranes (purple arrow) and intraretinal edema with neuroepithelial layer disorganization (purple asterisk). (B) (Second visit): B-scan ultrasonography of the right eye demonstrating a moderate-hyperreflective vitreous mass (pink arrow), consistent with acute vitreous hemorrhage. (C) (Third visit): Macular OCT of the right eye showing partial resolution of vitreous hemorrhage with restored retinal layer visibility. Left eye OCT exhibits ectopic inner foveal layers (orange asterisk) due to anterior retinal proliferation traction. (D) (Fourth visit): FFA revealing extensive non-perfusion areas (red asterisk) in both eyes and foveal arteriovenous anastomoses (red circle) in the left eye. Macular OCT demonstrating bilateral persistent ectopic inner foveal layers and left foveal architectural disruption with an absent foveal pit (orange asterisk). (E) (Final visit): Fundus photographs of both eyes. Residual macular epiretinal membrane folds (blue circle), a small amount of hemorrhage (yellow arrow), and vascular sheathing in the right eye (green arrow). Regression of arteriovenous anastomoses in the left eye. FFA = fundus fluorescein angiography.

**Figure 2.**
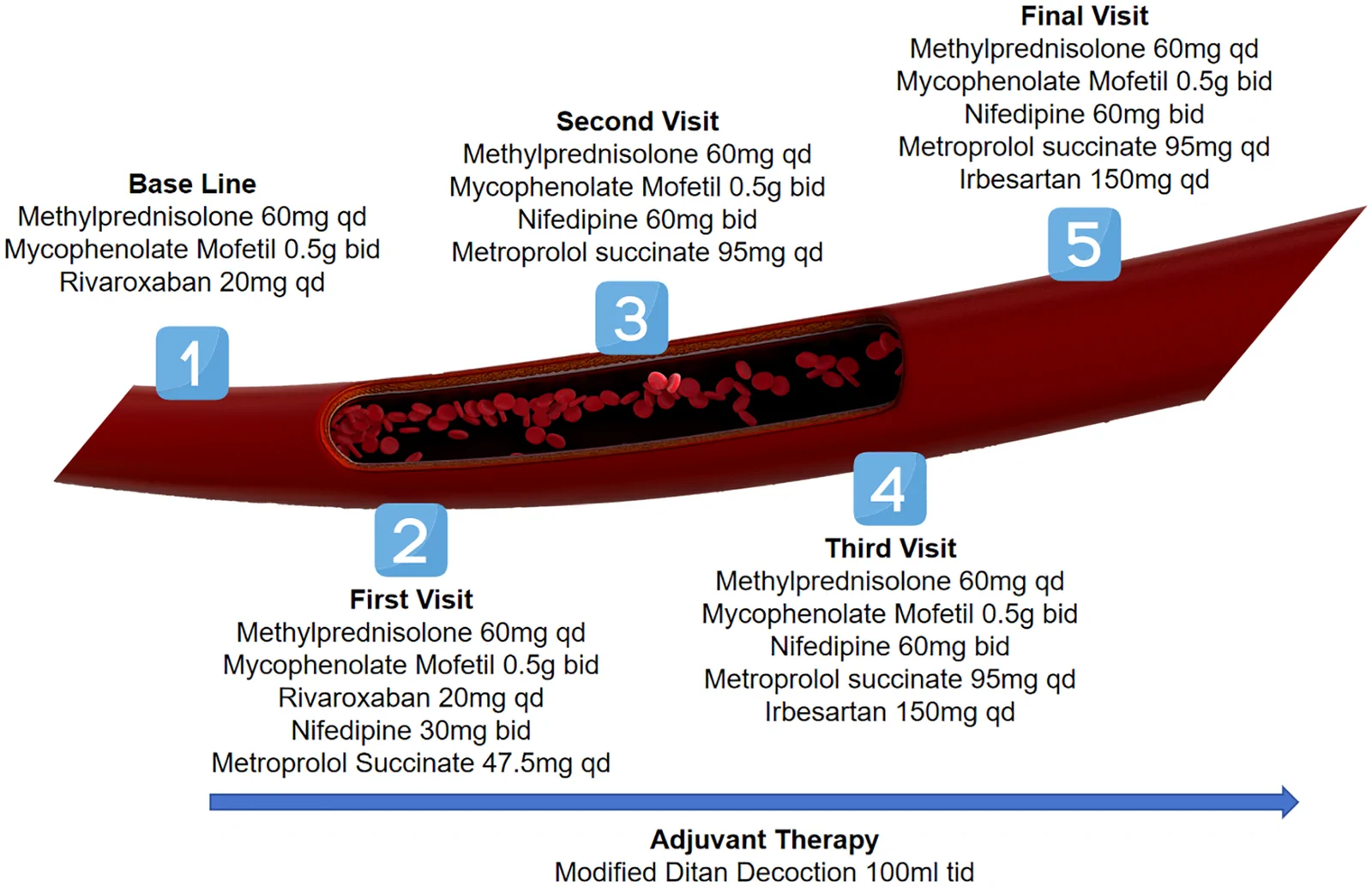
Treatment plan for the patient.

In response to these findings, the patient’s treatment regimen was supplemented with Nifedipine and Metoprolol Succinate to achieve optimal BP control. Additionally, dietary modifications were recommended, specifically reducing salt intake and incorporating daily low-intensity exercises such as brisk walking to support overall health. Furthermore, the patient underwent syndrome differentiation and personalized treatment planning grounded in the Four Diagnostic Methods of Traditional Chinese Medicine (TCM) (i.e., inspection, auscultation and olfaction, inquiry, and palpation). Subsequently, a tailored prescription of TCM was administered as an adjunctive therapeutic approach. Each daily dose of the TCM formulation was prepared through aqueous decoction to a standardized concentration, then fractionated into 3 equal aliquots for administration. The regimen required oral intake of each aliquot 30 minutes postprandially following breakfast, lunch, and dinner to ensure consistent systemic absorption. Figure 1 showcases the serial ocular examination results obtained during the patient’s visits, while Figure 2 outlines the adjustments made to the treatment regimen over time.

Two days after initiating the treatment regimen (second visit), the patient experienced an abrupt recurrence of decreased visual acuity in the right eye, accompanied by the appearance of floating red mist-like shadows in their visual field. Upon the second consultation, the patient’s BP was measured at 168/106 mm Hg, with visual acuity of the right eye at HM/20 cm and visual acuity of the left eye at 0.02. Examination revealed vitreous hemorrhage in the right eye, which obscured visualization of the fundus. B-scan ultrasonography demonstrated multiple dense, medium-sized reflective foci within the vitreous dark zone of the right eye, with positive posterior vitreous motion observed. Consequently, the rivaroxaban component of the treatment regimen was discontinued, and the dosage of antihypertensive medications was escalated, while all other facets of the treatment plan remained unaltered.

Two weeks later, at the third consultation, the patient’s BP was recorded at 154/99 mm Hg, with visual acuity of both eyes at counting fingers/30 cm. Compared to the previous examination, the vitreous hemorrhage in the right eye exhibited improved absorption. However, the retinal vessels surrounding the fovea of the left eye displayed tortuosity. The treatment plan was adjusted to further increase the dosage of antihypertensive medications and to incorporate supplementary treatment with TCM, based on syndrome differentiation and treatment principles.

One week thereafter, at the fourth consultation, the patient’s BP had decreased to 143/85 mm Hg, with visual acuity of the right eye improving to 0.08 and visual acuity of the left eye remaining at counting fingers/30 cm. The vitreous hemorrhage in the right eye showed marked absorption compared to previous examinations, but retinal folds were observed in the macular area. In the left eye, an arteriovenous anastomosis crossing the foveal center was visible. Fundus fluorescein angiography (FFA) of both eyes revealed extensive areas of non-perfused retina and fluorescein leakage in the macular regions. In light of these findings, the patient underwent fractional retinal laser photocoagulation. During this period, the patient’s TCM prescription was further personalized based on the adjustments made during the third consultation.

Six weeks later, at the final consultation, the patient’s BP was 142/88 mm Hg, with visual acuity of the right eye stable at 0.1 and visual acuity of the left eye remaining at 0.02. Fundus examination revealed evenly distributed panretinal photocoagulation scars in both eyes, and the caliber of the arteriovenous anastomosis in the left eye had narrowed compared with prior observations.

## 3. Discussion

SLE retinopathy occurs in 3% of patients with well-managed SLE and rises to 29% in those with severe disease manifestations.^[18]^ The predominant feature of SLE retinopathy is microvasculopathy, characterized by retinal hemorrhages and cotton-wool spots that account for 80% of cases and generally exhibit spontaneous resolution with a benign prognosis.^[19]^ Antiphospholipid syndrome–related vaso-occlusive retinopathy constitutes another well-recognized ocular complication of SLE. Antiphospholipid antibodies are detectable in approximately 29% of patients with SLE, and among those with persistently positive antibodies who fulfill the clinical criteria for antiphospholipid syndrome, the incidence of retinopathy reaches approximately 25%.^[20]^ This condition typically manifests as occlusion of the major retinal vessels with a pronounced thrombotic tendency.^[21]^Conversely, severe vaso-occlusive retinopathies, notably including Purtscher-like retinopathy, constitute a distinctive and uncommon subset within the spectrum of SLE retinopathies. Patients affected by these disorders tend to be younger, frequently under 25 years of age, have a shorter duration of SLE, demonstrate heightened disease activity, and experience a worsened visual prognosis.^[22]^ Studies have elucidated that the defining characteristics of severe vaso-occlusive retinopathies primarily encompass capillary occlusion and diffuse capillary non-perfusion.^[10]^ Notably, a striking 75% of SLE patients with Purtscher-like retinopathy exhibit CNS involvement, representing approximately a threefold increase compared to SLE patients without ocular fundus lesions.^[23]^ The microscopic brain manifestations observed in SLE patients with CNS involvement, which share similarities with those seen in Purtscher-like retinopathy, may be attributed to microvascular lesions stemming from complement activation, thereby providing a plausible explanation for the correlation between these 2 entities.^[24]^

A comprehensive review of the literature reveals an absence of universally accepted standard treatment protocols for Purtscher-like retinopathy, as well as a lack of therapeutic or interventional strategies that are universally applicable to all instances of this condition. It is worth highlighting that prior to the widespread adoption of steroid therapy, more than half of SLE patients developed retinopathy.^[25]^ However, with the advent and application of steroids and immunosuppressive therapies, the incidence of retinopathy has dramatically decreased, ranging from 3% in well-controlled disease to 29% in active multisystem disease.^[18]^ Despite this patient having received standard SLE treatment with steroids and immunosuppressants prior to presenting to the ophthalmology department, unfortunately, their ocular condition remained incompletely controlled.

Following the initial diagnosis in our hospital, the patient developed a dense vitreous hemorrhage in the right eye. We attributed this event to retinal neovascularization and capillary non-perfusion, exacerbated by severe hypertension; concurrent anticoagulation likely aggravated the vascular leakage. Accordingly, antihypertensive therapy was initiated, and anticoagulation was discontinued. However, persistent hypertension and poor fundus visualization precluded timely FFA at the second visit. By the third visit, the vitreous hemorrhage had partially cleared without rebleeding, obviating the need for vitrectomy. Nevertheless, ongoing BP elevation and media opacity prompted further intensification of antihypertensive treatment and additional deferral of FFA. At the fourth visit, BP was adequately controlled, and the fundus became clearly visible. FFA confirmed extensive retinal capillary non-perfusion in the right eye, as previously suspected. Moreover, the ischemic drive had fostered arteriovenous anastomoses in the left eye that traversed the foveal center, further compromising visual function. At the final follow-up, bilateral panretinal photocoagulation had been performed, ameliorating retinal ischemia. Consequently, the left eye anastomotic vessels partially regressed, and visual acuity improved marginally in both eyes.

Purtscher-like retinopathy in the setting of SLE remains scarcely reported in the published literature. Among the limited documented cases, standard therapeutic regimens have largely relied on topical corticosteroids and anti-vascular endothelial growth factor agents, with overall suboptimal clinical outcomes.^[22]^ Against this background of limited effective treatments, we administered modified Ditan Decoction as an adjunctive therapy in the present case. Across 4 serial follow-up visits, the patient’s BP was gradually stabilized. However, the risk of hemorrhage secondary to neovascularization within retinal non-perfusion zones remained critically elevated. This persistent, unmitigated risk accounted for the vitreous hemorrhage observed at the second visit. Encouragingly, no further episodes of intraocular hemorrhage occurred in either eye for the remainder of the follow-up period, providing indirect evidence that adjunctive modified Ditan Decoction may have contributed to the patient’s clinical trajectory.

A growing body of preclinical and clinical evidence has corroborated the therapeutic potential of Ditan Decoction across a spectrum of neurodegenerative and neurovascular disorders, including Alzheimer’s disease,^[26]^ ischemic and hemorrhagic stroke,^[27–29]^ and vascular dementia.^[30]^ Emerging data suggest its multifaceted neuroprotective mechanisms of this herbal formulation may involve: 1) modulation of the PI3K-Akt-HIF1α signaling pathway to suppress neuronal apoptosis and downstream oxidative stress cascades; 2) attenuation of both systemic and local neuroinflammatory responses via targeted regulation of proinflammatory cytokine networks; 3) Inhibition of ferroptotic cell death pathways in metabolically vulnerable neural tissues.^[29,30]^ Given the embryological and anatomical continuity between the retina and the CNS via the optic nerve, we postulate that Ditan Decoction exerts parallel protective effects on retinal tissue through these coordinated neuroprotective and vasculoprotective pathways. Nevertheless, the precise molecular and cellular mechanisms underlying the observed ocular therapeutic benefits remain incompletely elucidated and warrant further rigorous investigation.

## 4. Conclusion

Given the heterogeneous, multisystem nature of SLE and its broad spectrum of clinical manifestations, selection of the appropriate specialty for initial patient presentation is a critical determinant of timely diagnosis and optimal disease management. Rheumatology and dermatology clinics represent the most common points of first contact for patients with SLE, and thus bear primary responsibility for early diagnostic evaluation and initial care delivery. Although Purtscher-like retinopathy occurs far less frequently than cutaneous manifestations of SLE, its potential to cause permanent visual impairment merits close attention and poses distinct challenges to early recognition and intervention when patients first present to non-ophthalmology settings.

In the present case, the patient initially presented with blurred vision and fever, yet did not receive a comprehensive ophthalmologic evaluation at the first encounter. This clinical picture was suggestive of macular foveal involvement: a region supplied predominantly by the choroidal circulation: indicating that the patient’s early symptoms likely stemmed from chorioretinal vasculitis. The choroidal and retinal microvasculature shares key structural and pathophysiological features with renal micro vessels, as both are highly susceptible to immune complex deposition in the context of autoimmune disease. Notably, the ocular fundus harbors the only directly observable microvascular bed in the human body; as such, alterations in fundus vessels frequently precede or develop in parallel with microvascular pathology in other systemic organs. Accumulating evidence further underscores that changes in retinal capillary architecture closely mirror disease activity, progression, and remission in systemic inflammatory disorders.^[31,32]^

Accordingly, careful screening for ocular symptoms at the initial consultation is essential, and a multidisciplinary collaborative care model should be prioritized to enable early detection of ocular microvascular injury. Timely targeted intervention is critical to prevent irreversible vision loss and other severe ocular complications. When ocular symptoms or suspected ocular involvement are identified at initial presentation in rheumatology or dermatology settings, prompt referral to ophthalmology for specialist assessment is warranted. In turn, ophthalmologists should maintain close liaison with rheumatology and dermatology teams to jointly monitor disease activity, facilitate real-time clinical information exchange, and deliver coordinated, patient-centered care. Beyond standard therapeutic regimens, adjunctive TCM interventions may be considered when clinically appropriate, as a complementary strategy to support favorable disease outcomes.

## 5. Limitation

This study exclusively documents the diagnostic and therapeutic trajectory of a single patient, thereby lacking the generalizability achievable through control group comparisons or larger sample validation to substantiate the conclusions. Second, the unique coexisting comorbidities of the patient may constrain the applicability of our findings to other clinical populations. Notwithstanding the constraints of sample size, this case constitutes the inaugural report documenting successful application of TCM therapy in patients with SLE complicated by Purtscher-like retinopathy. The potential therapeutic benefits for analogous patient cohorts in future clinical practice warrant careful consideration.

## Author contributions

**Conceptualization:** Kaiyao Chi.

**Data curation:** Kaiyao Chi, Tong Guo.

**Investigation:** Kaiyao Chi, Xiaoyang Xie.

**Methodology:** Kaiyao Chi, Tong Guo.

**Software:** Xiaoyang Xie.

**Validation:** Xiaoyang Xie.

**Visualization:** Xiaoyang Xie.

**Formal analysis:** Tong Guo.

**Funding acquisition:** Daoxi Lei, Huili Li.

**Project administration:** Daoxi Lei.

**Resources:** Daoxi Lei, Huili Li.

**Supervision:** Huili Li.

**Writing – review & editing:** Xiaoyang Xie, Tong Guo, Daoxi Lei, Huili Li.

**Writing – original draft:** Kaiyao Chi.
